# A New Method of Feeding in Cases of Intubation of the Larynx*Read before the Chicago Medical Society, September 3, 18.

**Published:** 1888-10

**Authors:** W. E. Casselberry

**Affiliations:** Professor of Materia Medica and Therapeutics and of Laryngology and Rhinology in the Chicago Medical College, Medical Department of the Northwestern University; 70 Monroe Street


### A NEW METHOD OF FEEDING IN CASES OF INTUBATION OF THE LARYNX BY POSITION, HEAD DOWNWARD, ON AN INCLINED PLANE.*

BY W. E. CASSELBERRY, M. D.,

Professor of Materia Medica and Therapeutics and of Laryngology and Rhinology in the Chicago Medical College, Medical Department of the Northwestern University.

The operation of intubation of the larynx, for the relief of diphtheritic croup and other forms of laryngeal stenosis, has become so familiar to all that it is unnecessary to review it, even in brief. We therefore proceed at once to consider the subject-proper of this report, which is the subsequent feeding of the patient. This is the most serious difficulty pertaining to the after-treatment. The tube has been inserted, the dyspnœa is relieved, and the child, previously much exhausted by its struggle for breath, usually rests and sleeps quietly for a time. All goes well for six hours or so, when comes the question of how to give nourishment.

It is manifest that, with the action of the epiglottis impaired by the open tube in position, fluids and even semi-solids in process of deglutition will run through the tube into the trachea and thence into the lungs. Pulmonary inflammation, especially recognized by the Germans as *schluck-pneumonie*, is thus excited, and this, under the circumstances, is nearly always fatal. Not that the entrance of food material into the lungs is the sole cause of pneumonia occurring in the course of diphtheritic laryngitis, for this complication frequently arises by simple extension of the diphtheritic inflammation downward, and occurs, at times, after tracheotomy, when deglutition is unimpaired, but it is an additional exciting factor and one which is capable of originating pneumonia in the absence of other causes.

It has been sought to obviate the trouble with the administration of liquids, by substituting semi-solids and ice in lieu of water, but these do not suffice. It is said, give the patients ice, wrapped in a cloth, to suck, and they will obtain sufficient water to supply the demands of the body, and this would seem to be true, but it is not, for they will cry piteously for water after sucking ice for hours.

The constant cry of water! water! is often so distressing as to cause both the friends of the patient and the surgeon to regret having selected this operation in preference to tracheotomy. The patient has fever and constant thirst, and even in sleep and in delirium will mutter and cry for water. The nasal feeding-tube was introduced to meet this necessity. A soft rubber catheter, which is attached to a Davidson syringe or to a fountain apparatus, is passed through the nostril into the stomach, and through it water, milk, and

---

* Read before the Chicago Medical Society, September 3, 18 .

other liquids can be rapidly introduced. It usually works well in the beginning before the child has learned what it is, but after the first time or two there is always an exhausting struggle, and one must also work very quickly to pump sufficient liquid into the stomach before the child will vomit tube and all, from the irritation of its presence in the fauces. Moreover, it is practically impossible to introduce the tube often enough to satisfy the desire for drink. So this method, although useful, is insufficient.

The artificial rubber epiglottis proved a failure. To further the solution of the problem Dr. Waxham devised the metal epiglottis tube, which is fitted at its top with a thin metal lid—the epiglottis, which, by means of a fine gold spring, is maintained in an upright position, except when the natural epiglottis pushes it down in process of swallowing. This tube works properly only when it is in exact position; if it becomes somewhat turned on its axis, or if it is small enough to sink a trifle low, leakage into the tube will ensue, through failure of the natural epiglottis to approximate accurately the tube and its metal lid. I feel constrained, also, while much admiring the ingeniousness of its mechanism, to advise against the use of the metal epiglottis tube, except, possibly, by the most skillful hands, because of the serious complication which the lid presents to the introduction of the extractor for withdrawal of the tube.

In June, 1888, through the courtesy of Dr. Frank Cary, I performed the operation of intubation of the larynx in a case of diphtheritic croup. We encountered the difficulties which I have enumerated, and which I had so frequently experienced before. The child was given ice, and still there was an incessant cry for water. The nasal feeding-tube was used, with the usual partial degree of success only. We were much distressed about the case, and in thinking of some means to administer liquid to the child, the thought occurred to me, Is there nothing in *position* that will help us? Answered at once by a second thought, Why yes! Stand the child on its head and let it drink. Certainly, by means of muscular action it could swallow upward through the œsophagus, just as one does when leaning far over to drink from a spring, while, in this position, the liquid could not gravitate upward through the tube. Naturally the idea followed that a modification of the position would suffice, one with the body inclined, head downward, at such an angle as to prevent water by gravitation, aided by any slight propulsive force given it in pharyngeal deglutition, from trickling through the tube. On going to the house, I met Dr. Cary and told him that I had determined to stand the child on its head and let it drink, when he remarked that the same thought had occurred to him; that he had even mentioned it that morning to another physician, who had laughed at the idea, but that he meant to suggest it to me. We directed the nurse to hold the child in her arms, on its back, with its legs tilted upward and its head hanging downward over her arm, so as to incline the body, neck, and tube at a considerable angle. In this position it would suck through a rubber tube from a glass, and swallow without the slightest difficulty all the liquid it wanted.

The matter seems so simple that we marvel that it had not been thought of previously. Several thousand cases have now been treated by intubation, and in all the necessity for a method such as this must have been apparent. Communication was held with Dr. F. E. Waxham, well known in connection with intubation, who said he had never heard of the method, and that he would try it. He has since done so in several cases, and he is enthusiastic over its success; says it is the next thing to the discovery of the operation itself. Dr. Cary communicated with Dr. O'Dwyer, who had heard nothing of it and seemed to ridicule the idea of the practicability of the method. Danger of congestion of the brain would occur to everyone, but it is not realized in practice.

The first case died from extension of membrane below the tube, but died having demonstrated the utility of this plan of feeding.

The second case in which I tried the method was one in the last extremity of diphtheritic croup, and seen through the courtesy of Dr. Frank Billings. A request for an hour's delay to send for apparatus was met by Dr. Billings' reply that the child would certainly then be dead. A *metal epiglottis tube* was inserted at once, and a good case thus presented to test the efficacy of the lid alone. In the upright position, with the epiglottis tube *in situ*, the patient could not swallow water or milk without coughing, indicating entrance into the trachea. In the inclined-plane position, with the head hanging downward, it took any quantity of liquid without the slightest difficulty. That child recovered.

The third case, left temporarily in my care by Dr. Waxham, was one of laryngeal growth, in which a tube had been inserted to relieve the dyspnœa. It also was wearing the epiglottis tube. It could swallow, but only with difficulty, and Dr. Waxham had already introduced the inclined-plane method, which was used with delight by the child, and much to the relief of the parents. It continued to work successfully under my observation as long as the tube was worn.

Regarding the exact position; the angle has varied in different cases, but from 45° to 90° seems necessary to obtain the best results. The child is held on its back in the arms of the nurse, the legs elevated, and the head left to hang over the arm. Then it may take the mouth of the feeding-bottle, suck through a tube from a glass or feed from a spoon. The only difficulty is encountered when the child is again placed in the upright position, which posture it must not be permitted to regain until it has been made to swallow three or four times after the vessel of liquid has been taken from its mouth, in order to swallow all the fluid which has gravitated into the pharynx and naso-pharynx. After they have learned this they will readily swallow several times, so as to force the liquid remaining in the throat into the stomach before the upright position is again taken, and then there is no trouble. The patient can be inclined without inconvenience for a minute or more, although much less than this only is necessary.

There is no danger of the tube slipping out unless one of too small size has been inserted, when it would become a fortunate accident, permitting the selection of a proper size for re-introduction.

70 Monroe Street.

